# Hematologic breast malignancies: a single-institution cohort study of 24 patients diagnosed between January 2008 and June 2023, with clinicopathological and outcome analysis

**DOI:** 10.3389/fonc.2026.1925669

**Published:** 2026-10-21

**Authors:** Lucia Navarro, María de la Haba, Pablo Flores-Paco, Víctor Atance, Francisco José Jiménez Nájar, Sergio Haro-Yuste, Fernando Leiva-Cepas, Concepción Herrera Arroyo, Cristina Morales-Estevez, Enrique Aranda, Juan de la Haba-Rodríguez

**Affiliations:** 1 Department of Medical Oncology, University Hospital Reina Sofía, Córdoba, Spain; 2 Maimonides Biomedical Research Institute (IMIBIC), Córdoba, Spain; 3 University of Córdoba, Córdoba, Spain; 4 Department of Hematology, University Hospital Virgen Macarena, Seville, Spain; 5 Department of Hematology, University Hospital Reina Sofía, Córdoba, Spain; 6 Department of Pathology, University Hospital Reina Sofía, Córdoba, Spain

**Keywords:** breast lymphoma, DLBCL, extranodal lymphoma, hematologic breast malignancy, MALT lymphoma, primary breast lymphoma

## Abstract

Hematologic breast malignancies (HBM) are rare and heterogeneous neoplasms that account for less than 1% of breast malignancies and may clinically and morphologically mimic primary breast carcinoma. We retrospectively analyzed 24 patients diagnosed with HBM at a single tertiary institution between January 2008 and June 2023 to describe their clinicopathological characteristics, treatment patterns and outcomes. The mean age at diagnosis was 60.3 ± 19.8 years, and the median age was 60.5 years (IQR 53.0–75.5); 23 patients (95.8%) were women. Diffuse large B-cell lymphoma was the most frequent subtype (n=8, 33.3%), followed by MALT lymphoma (n=5, 20.8%). Based on the clinical and staging information retrospectively available, 21 cases (87.5%) were classified as primary breast involvement and 3 (12.5%) as secondary involvement. All diagnoses were established by image-guided core needle biopsy. Systemic chemotherapy was administered to 19 patients (79.2%), radiotherapy to 9 (37.5%), and mastectomy to 2 (8.3%); the remaining 22 patients were managed without breast resection. After a crude median observed duration of 86.9 months (range 7.9–217.6), 11 patients (45.8%) had experienced progression or relapse and 13 (54.2%) had died. Only 3 deaths were attributed to progression of the hematologic malignancy. Median overall survival was 115.7 months (95% CI 54.9–not reached), whereas disease-specific survival was not reached. These findings highlight the importance of core needle biopsy and appropriate hematolymphoid lineage assessment in avoiding unnecessary breast surgery. Outcomes should be interpreted descriptively given the small, histologically heterogeneous cohort and the limitations inherent to its retrospective design.

## Introduction

The breast is an uncommon site for hematologic malignancies. Primary breast lymphoma (PBL), defined as the involvement of breast parenchyma in the absence of disseminated systemic lymphoma at diagnosis, accounts for approximately 0.4–0.7% of all non-Hodgkin lymphomas (NHL) and less than 1% of all breast malignancies (1, 2). Secondary breast involvement, occurring in patients with known systemic hematologic disease, is equally rare. Together, these entities—collectively termed hematologic breast malignancies (HBM)—present a distinct diagnostic and therapeutic challenge to clinicians, radiologists, and pathologists.

From a clinical perspective, HBM frequently mimics primary breast carcinoma. Both entities commonly present as a palpable mass or a mammographic density, with non-specific imaging features on ultrasound and MRI that rarely allow radiological distinction (3). At the histopathological level, certain lymphoma subtypes—most notably DLBCL and small B-cell lymphomas with a single-file infiltrative pattern—may closely resemble invasive lobular carcinoma, particularly when lymphoma markers are not included in the initial immunohistochemical panel (4, 5). In some exceptional cases, lymphomas have been reported to express hormone receptors, further complicating the differential diagnosis (4). The clinical consequences of misclassification are significant: unlike carcinomas, most HBMs do not require mastectomy or axillary dissection, and subjecting a patient to unnecessary surgery for a lymphoma constitutes avoidable morbidity (1, 6).

Despite growing awareness of this entity, the published evidence base remains fragmented. The majority of existing studies comprise case reports, small retrospective series, or registry-based analyses focused predominantly on DLBCL (7, 8). Large contemporary series examining the full histological spectrum of HBM—including both lymphomas and leukemic infiltrates—and providing long-term survival data stratified by subtype are scarce, particularly from European centers (6, 9). This scarcity limits the development of standardized diagnostic and management guidelines.

The present study addresses this gap by reporting on 24 patients with HBM diagnosed and followed over 15 years at a single Spanish tertiary referral center. Our objectives were to: (i) characterize the clinical and pathological features of HBM across the full subtype spectrum; (ii) describe treatment patterns in the context of current hematologic oncology practice; and (iii) provide subtype-stratified survival and outcome data to complement the existing literature on this rare disease.

## Methods

### Study design and setting

We conducted a retrospective observational cohort study at University Hospital Reina Sofía, a tertiary referral center in Córdoba, Spain, affiliated with the Maimonides Biomedical Research Institute (IMIBIC). The study covered the period from January 2008 to June 2023. The institutional ethics committee approved the study protocol, and all procedures conformed to the Declaration of Helsinki. Given the retrospective nature of the study, individual informed consent was waived in accordance with applicable national regulations.

### Patient selection

Patients were identified through a systematic search of the institutional pathology and oncology electronic databases. Inclusion criteria were: (1) histopathological confirmation of a hematologic malignancy involving the breast, whether primary or secondary; (2) complete clinical, imaging, and pathology records; and (3) available follow-up data. Patients with insufficient tissue for immunohistochemical analysis or incomplete medical records were excluded. A total of 24 patients fulfilled all criteria and constitute the study cohort.

Primary breast involvement was classified retrospectively using the Wiseman–Liao framework (11), considering the available clinical presentation and staging information. Ipsilateral axillary nodal involvement was permitted, while cases with evidence of systemic hematological disease involving the breast were classified as secondary breast involvement.

### Data collection

Data were extracted from electronic medical records by two independent investigators (LN, MdlH) and verified against pathology and radiology reports. Variables collected included: age at diagnosis, sex, menopausal status, mode of detection, presence of B symptoms (fever, night sweats, unintentional weight loss), personal history of hematologic malignancy, histological subtype according to the 2022 WHO Classification of Hematolymphoid Tumors (10), disease extent (breast only versus breast plus axillary lymph node involvement), Ann Arbor staging adapted for extranodal sites (IE or IIE), tumor size on imaging, hormone receptor expression (estrogen receptor [ER] and progesterone receptor [PR] by immunohistochemistry), treatment modalities (chemotherapy regimen, radiotherapy, surgery), disease progression or relapse with date, vital status, and date of death.

Data on bone marrow biopsy, staging modality (PET-CT versus CT), LDH, ECOG performance status, IPI and CNS-IPI, laterality and bilaterality, Ki-67 index, cell of origin by the Hans algorithm, FISH results, and HIV or immunosuppression status were not recorded consistently across the accrual period and could not be retrieved retrospectively for the full cohort, despite a dedicated review of the clinical records. This reflects the span of the series, over which institutional documentation practice and the standard diagnostic work-up for extranodal lymphoma changed substantially. These variables are reported as not available rather than as negative findings.

### Histopathological evaluation

Pathological evaluation included hematoxylin and eosin (H&E) staining and immunohistochemical markers selected according to the morphological and diagnostic context, including CD20, CD3, CD10, BCL2, BCL6, MUM1 and Ki-67. Additional markers, including cyclin D1, TdT, CD5, CD23, CD138 and CD56, were applied selectively. ER and PR results were reviewed when available in the pathology documentation, although a reliable testing denominator and the original indication for testing could not be retrospectively reconstructed for every case. For the purposes of this study, the available pathological information was reassessed according to the 5th edition of the WHO Classification of Hematolymphoid Tumors (WHO-HAEM5), with reference to the International Consensus Classification (ICC) 2022. This reassessment resulted in the reclassification of two cases relative to their original diagnostic categorization. Progression and relapse events were retrospectively reassessed from the available clinical and radiological documentation using the principles of the Lugano 2014 criteria whenever sufficient information was available (12). Representative histopathological images with immunostaining are shown in **Supplementary Figure 1**.

### Statistical analysis

Descriptive statistics were used to summarize patient and disease characteristics. Continuous variables are presented as mean ± standard deviation (SD), median with interquartile range (IQR), or median with range, as appropriate; categorical variables are reported as absolute numbers and percentages. Overall survival (OS) was defined as the interval from diagnosis to death from any cause, with patients alive censored at the date of their last documented clinical contact. Disease-specific survival was defined as the interval from diagnosis to death attributed to progression of the hematological malignancy; deaths from other causes were censored at the date of death. Time to progression (TTP) was defined as the interval from diagnosis to the first documented disease progression or relapse. Overall and disease-specific survival were estimated using the Kaplan–Meier method. The median potential follow-up was estimated using the reverse Kaplan–Meier method. Patient accrual ended in June 2023, whereas vital status and follow-up were updated using the most recent clinical information available in 2026. Given the small and histologically heterogeneous cohort, all analyses were considered descriptive. Analyses were performed using IBM SPSS Statistics version 25 (IBM Corp., Armonk, NY, USA).

## Results

### Patient and disease characteristics

Twenty-four patients were identified (23 women, 95.8%; mean age 60.3 ± 19.8 years, median 60.5, IQR 53.0–75.5, range 17–93) (**Figure 1A**). All diagnoses were established by image-guided core needle biopsy. The most prevalent subtype was diffuse large B-cell lymphoma (DLBCL, n=8, 33.3%), followed by MALT lymphoma (n=5, 20.8%), follicular lymphoma (n=3, 12.5%) and CLL/SLL (n=3, 12.5%), with nodal marginal zone lymphoma (n=2), B-lymphoblastic lymphoma/leukemia (n=2) and Burkitt lymphoma (n=1) completing the spectrum (**Table 1**).

**Table 1:** Clinicopathological characteristics of patients with hematologic breast malignancies (n=24).

Variable	n (%) or value	Range / notes
Demographics		
Total patients	24 (100%)	
Female sex	23 (95.8%)	
Male sex	1 (4.2%)	
Age at diagnosis, years — mean ± SD	60.3 ± 19.8	Range 17–93
Age at diagnosis, years — median (IQR)	60.5 (53–75.5)	
Histological subtype (Who-HAEM5)		
DLBCL	8 (33.3%)	
MALT lymphoma	5 (20.8%)	
Follicular lymphoma	3 (12.5%)	
CLL/SLL	3 (12.5%)	
Nodal marginal zone lymphoma	2 (8.3%)	
B-lymphoblastic lymphoma/leukaemia	2 (8.3%)	
Burkitt lymphoma	1 (4.2%)	
Clinical presentation		
B symptoms	2 (8.3%)	
Detected by mammography	11 (45.8%)	
Palpable breast mass	8 (33.3%)	
Incidental / follow-up imaging	5 (20.8%)	
Disease characteristics		
Breast parenchyma only (no axillary involvement)	12 (50.0%)	Anatomical extent
Breast + axillary lymph node involvement	12 (50.0%)	Anatomical extent
Ann Arbor stage IE	11 (45.8%)	Reported separately from nodal status
Ann Arbor stage IIE	13 (54.2%)	Includes 1 case with regional non-axillary involvement
Tumour size on imaging, cm — mean ± SD	2.5 ± 1.8	n=21 evaluable; range 0.9–8
ER and/or PR positivity among cases with available testing	0	Testing denominator could not be reliably reconstructed
Prior history of haematologic malignancy	6 (25.0%)	
Primary breast involvement (Wiseman–Liao)	21 (87.5%)	
Secondary breast involvement	3 (12.5%)	
Treatment		
Systemic treatment	19 (79.2%)	
Radiotherapy	9 (37.5%)	
Mastectomy	2 (8.3%)	Both patient-driven
No breast surgery	22 (91.7%)	
Outcomes		
Disease progression / relapse	11 (45.8%)	
Death (any cause)	13 (54.2%)	
— Disease progression	3 (23.1%)	of 13 deaths
— Unrelated cause	6 (46.2%)	of 13 deaths
— Second neoplasm	2 (15.4%)	of 13 deaths
— Treatment toxicity	1 (7.7%)	of 13 deaths
— Not recorded	1 (7.7%)	of 13 deaths
Alive without evidence of disease	5 (20.8%)	
Alive with disease	6 (25.0%)	
Observation time, months — median	86.9	Range 7.9–217.6
Reverse Kaplan-Meier follow-up, months	159.7	Potential follow-up estimate

DLBCL, diffuse large B-cell lymphoma; MALT, mucosa-associated lymphoid tissue; CLL/SLL, chronic lymphocytic leukaemia/small lymphocytic lymphoma; ER, oestrogen receptor; PR, progesterone receptor; IHC, immunohistochemistry; IQR, interquartile range.

Anatomical extent and Ann Arbor stage are reported as separate variables and are not interchangeable. Each patient was censored at death or at the date of last documented follow-up. Only 3 of the 13 deaths were attributable to disease progression; disease-specific and overall survival are therefore not equivalent and are reported separately.

Twelve patients (50.0%) had ipsilateral axillary nodal involvement. Anatomical extent and Ann Arbor stage were analyzed as separate variables. Eleven patients (45.8%) were classified as stage IE and 13 (54.2%) as stage IIE. One patient classified as stage IIE had regional non-axillary nodal involvement without axillary involvement.

Six patients (25.0%) had a documented previous hematological malignancy. Based on the clinical presentation and staging information retrospectively available, 21 cases (87.5%) were classified as primary breast involvement and 3 (12.5%) as secondary involvement. Previous history of a hematological malignancy and classification as primary or secondary breast involvement were considered separate variables.

Mean tumor size was 2.5 ± 1.8 cm (n=21 evaluable). Systemic treatment was administered to 19 patients (79.2%) and radiotherapy to 9 (37.5%); only 2 (8.3%) underwent mastectomy, both at their own request. Twenty women were postmenopausal, three premenopausal, and menopausal status was not applicable in the single male patient. After a median observation time of 86.9 months (range 7.9–217.6), progression or relapse had occurred in 11 patients (45.8%) and 13 (54.2%) had died, of whom 3 (23.1% of deaths) died of disease progression. Median overall survival was 115.7 months (95% CI 54.9 – not reached), whereas median disease- specific survival was not reached, with an estimated 86.8% at 15 years.

### Histological subtypes and disease characteristics

The histological spectrum encompassed seven distinct entities, as shown in **Figure 2**. DLBCL was the most frequent diagnosis, accounting for 8 cases (33.3%), followed by MALT lymphoma (n=5, 20.8%), follicular lymphoma (n=3, 12.5%), and CLL/SLL (n=3, 12.5%). Less common subtypes included nodal marginal zone lymphoma (n=2, 8.3%), B-lymphoblastic lymphoma/leukemia (n=2, 8.3%), and Burkitt lymphoma (n=1, 4.2%). No myeloid malignancies, breast implant-associated anaplastic large cell lymphomas (BIA-ALCL), T-cell lymphomas or plasma-cell neoplasms were identified. None of the patients had breast implants.

Anatomical extent and Ann Arbor stage are reported separately. Twelve patients (50.0%) had disease confined to the breast parenchyma and twelve (50.0%) had concurrent ipsilateral axillary lymph node involvement (**Figure 1C**). By Ann Arbor staging adapted for extranodal sites, 11 patients (45.8%) were stage IE and 13 (54.2%) stage IIE; one stage IIE case had no axillary involvement, reflecting regional non-axillary nodal disease. Mean tumor size on imaging was 2.5 ± 1.8 cm (range 0.9–8.0 cm) among the 21 patients with a recorded measurement. Neither ER nor PR was expressed in any tested case.

**Figure 1:**
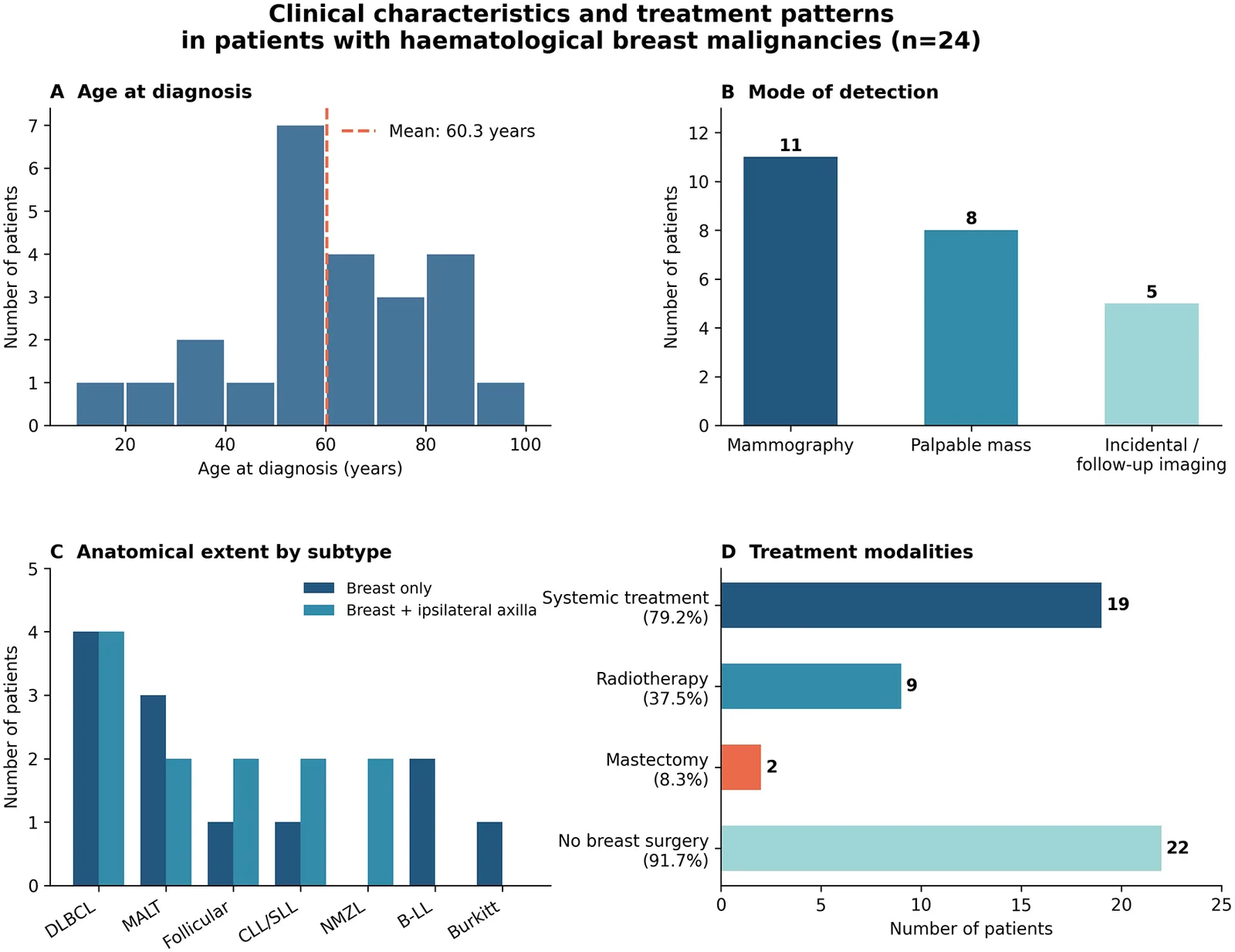
Clinical characteristics and treatment patterns among 24 patients with hematological breast malignancies. **(A)** age distribution at diagnosis (mean 60.3 years; range 17–93). **(B)** mode of detection (mammography, n=11, 45.8%; palpable breast mass, n=8, 33.3%; incidental or follow-up imaging, n=5, 20.8%). **(C)** anatomical extent according to histological subtype, distinguishing breast-only involvement (n=12, 50.0%) from breast plus ipsilateral axillary involvement (n=12, 50.0%). Anatomical extent was analyzed separately from Ann Arbor stage. **(D)** treatment modalities administered, including systemic treatment (n=19, 79.2%), breast-directed radiotherapy (n=9, 37.5%), mastectomy (n=2, 8.3%), and management without breast surgery (n=22, 91.7%). DLBCL, diffuse large B-cell lymphoma; MALT, mucosa-associated lymphoid tissue lymphoma; CLL/SLL, chronic lymphocytic leukemia/small lymphocytic lymphoma; NMZL, nodal marginal zone lymphoma; B-LL, B-lymphoblastic lymphoma/leukemia.

**Figure 2:**
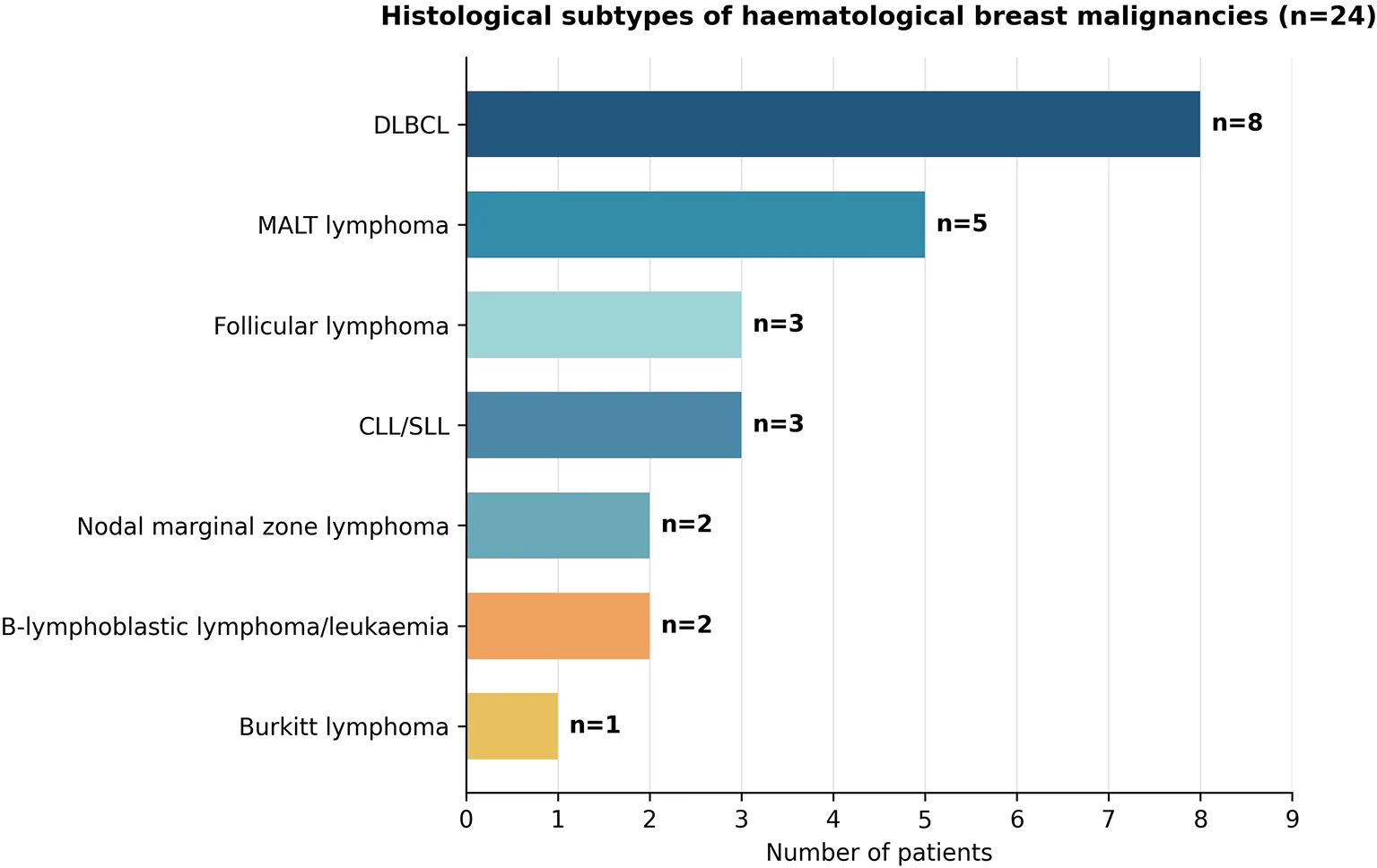
Distribution of histological subtypes among 24 patients with hematological breast malignancies. The horizontal bar chart shows the absolute number of cases corresponding to each histological entity. DLBCL was the most frequent subtype (n=8, 33.3%), followed by MALT lymphoma (n=5, 20.8%), follicular lymphoma (n=3, 12.5%), CLL/SLL (n=3, 12.5%), nodal marginal zone lymphoma (n=2, 8.3%), B-lymphoblastic lymphoma/leukemia (n=2, 8.3%), and Burkitt lymphoma (n=1, 4.2%). DLBCL, diffuse large B-cell lymphoma; MALT, mucosa-associated lymphoid tissue lymphoma; CLL/SLL, chronic lymphocytic leukemia/small lymphocytic lymphoma.

### Treatment

Treatment modalities are summarized by histological subtype in **Table 2** and **Figure 1D**. Systemic treatment was administered to 19 patients (79.2%). DLBCL was principally treated with R-CHOP (rituximab, cyclophosphamide, doxorubicin, vincristine and prednisone), whereas patients with CLL/SLL received rituximab-based treatment. Patients with MALT lymphoma received rituximab monotherapy or rituximab–bendamustine according to disease extent and clinical context. Both patients with B-lymphoblastic lymphoma/leukemia and the patient with Burkitt lymphoma received intensive HyperCVAD-based induction chemotherapy. No patient was documented as having received prophylactic CNS-directed treatment.

**Table 2:** Disease extent, stage and treatment by histological subtype.

Histological subtype	n	Breast only	Breast + axilla	Stage IE	Stage IIE	Systemic treatment	Radiotherapy	Mastectomy
DLBCL	8	4/8	4/8	4	4	7 (87.5%)	5 (62.5%)	0 (0.0%)
MALT lymphoma	5	3/5	2/5	3	2	5 (100.0%)	1 (20.0%)	1 (20.0%)
Follicular lymphoma	3	1/3	2/3	0	3	1 (33.3%)	0 (0.0%)	0 (0.0%)
CLL/SLL	3	1/3	2/3	1	2	1 (33.3%)	1 (33.3%)	1 (33.3%)
Nodal marginal zone lymphoma	2	0/2	2/2	0	2	2 (100.0%)	1 (50.0%)	0 (0.0%)
B-lymphoblastic lymphoma/leukaemia	2	2/2	0/2	2	0	2 (100.0%)	1 (50.0%)	0 (0.0%)
Burkitt lymphoma	1	1/1	0/1	1	0	1 (100.0%)	0 (0.0%)	0 (0.0%)
TOTAL	24	12/24	12/24	11	13	19 (79.2%)	9 (37.5%)	2 (8.3%)

Column totals sum exactly to the cohort totals: chemotherapy 19/24 (79.2%), radiotherapy 9/24 (37.5%), mastectomy 2/24 (8.3%), stage IE 11 and stage IIE 13. One follicular lymphoma case classified as stage IIE had no axillary involvement, reflecting regional non-axillary nodal disease. One DLBCL patient aged 93 did not receive chemotherapy and was treated with radiotherapy alone.

Breast-directed radiotherapy was administered to 9 patients (37.5%), including 5 patients with DLBCL. Radiotherapy was used primarily as consolidation treatment in DLBCL or as definitive local treatment in selected patients with localized disease. Detailed information regarding radiotherapy dose, treatment fields and technique, number of systemic treatment cycles, formal response rates and rituximab maintenance was not available consistently across the 15.5-year retrospective study period.

Only 2 patients (8.3%) underwent mastectomy. In both cases, the decision was driven by patient preference following disclosure of the hematological diagnosis and multidisciplinary discussion rather than by a formal oncological indication. The remaining 22 patients (91.7%) were managed without surgical resection of the breast.

### Survival and outcomes

Outcome data are presented by histological subtype in **Table 3** and survival estimates in **Table 4**. After a median observation time of 86.9 months (range 7.9–217.6; reverse Kaplan-Meier estimate 159.7 months), progression or relapse had been documented in 11 patients (45.8%) and 13 patients (54.2%) had died. Cause of death was disease progression in 3 patients, an unrelated cause in 6, a second neoplasm in 2, and treatment toxicity in 1; in one case the cause was not recorded. At last follow-up, 5 patients (20.8%) were alive with no evidence of disease and 6 (25.0%) were alive with disease.

**Table 3:** Outcomes and survival by histological subtype.

Histological subtype	n	Deaths n (%)	Disease-related deaths	Progression / relapse n (%)	Median observation (mo)	Observation range (mo)	Median time to progression (mo)	Alive NED n (%)
DLBCL	8	6 (75.0%)	1	3 (37.5%)	66.6	11.2–170.1	14.5	2 (25.0%)
MALT lymphoma	5	1 (20.0%)	0	3 (60.0%)	85.4	47.8–131.8	43.4	1 (20.0%)
Follicular lymphoma	3	2 (66.7%)	0	1 (33.3%)	88.5	44.6–146.1	55.7	0 (0.0%)
CLL/SLL	3	2 (66.7%)	0	0 (0.0%)	120.5	107.3–159.7	—	1 (33.3%)
Nodal marginal zone lymphoma	2	0 (0.0%)	0	2 (100.0%)	200.1	182.6–217.6	136.2	0 (0.0%)
B-lymphoblastic lymphoma/leukaemia	2	2 (100.0%)	2	2 (100.0%)	29.1	7.9–50.3	21.9	0 (0.0%)
Burkitt lymphoma	1	0 (0.0%)	0	0 (0.0%)	78.2	78.2	—	1 (100.0%)
TOTAL	24	13 (54.2%)	3	11 (45.8%)	86.9	7.9–217.6	43.7	5 (20.8%)

NED, no evidence of disease. Median time to progression was calculated among the 10 patients with a dated progression event; "—" indicates no dated event in the subgroup. One additional patient had a documented progression event without a reliable event date. This event was included in the overall progression count but excluded from the descriptive time-to-progression calculation.

**Table 4:** Kaplan-Meier survival estimates.

Analysis	Median (months)	Estimated survival
Overall survival, whole cohort (13 events)	115.7 (95% CI 54.9–not reached)	5-yr 70.8% · 10-yr 46.5% · 15-yr 31.9%
Disease-specific survival, whole cohort (3 events)	Not reached	5-yr 86.8% · 10-yr 86.8% · 15-yr 86.8%
Overall survival, DLBCL (n=8, 6 events)	102.1	5-yr 62.5% · 10-yr 16.7%

The divergence between overall and disease-specific survival is the central outcome finding of this cohort: most patients died with, not of, their haematologic disease. Confidence intervals are wide given the number of events and should be interpreted accordingly.

Median overall survival for the whole cohort was 115.7 months (95% CI 54.9 – not reached), with estimated survival of 70.8% at 5 years, 46.5% at 10 years and 31.9% at 15 years. Median disease-specific survival was not reached, with an estimate of 86.8% at 5, 10 and 15 years. Among the 8 patients with DLBCL, 6 deaths were recorded and median overall survival was 102.1 months, although only one of these deaths was attributable to lymphoma progression. Both patients with B-lymphoblastic lymphoma/leukemia died of progressive disease, at 7.9 and 50.3 months. Both deaths in the CLL/SLL subgroup, and the single death among MALT lymphoma patients, were attributable to unrelated causes; no progression event was documented in the CLL/SLL subgroup. Median time to progression among the 10 patients with a dated event was 43.7 months.

Clinicopathological characteristics and outcomes according to primary versus secondary breast involvement are presented descriptively in **Table 5**. No formal statistical comparison was performed because only three cases were classified as secondary involvement.

**Table 5:** Characteristics and outcomes of primary versus secondary breast involvement.

Variable	Primary (n=21)	Secondary (n=3)
Demographics and presentation		
Female sex	20 (95.2%)	3 (100.0%)
Age, years — mean ± SD	62.4 ± 19.2	46 ± 22.1
Prior haematologic malignancy	3 (14.3%)	3 (100.0%)
B symptoms	2 (9.5%)	0 (0.0%)
Detected by mammography	8 (38.1%)	3 (100.0%)
Palpable mass	8 (38.1%)	0 (0.0%)
Disease characteristics		
Breast + axillary involvement	11 (52.4%)	1 (33.3%)
Ann Arbor stage IE / IIE	9 / 12	2 / 1
Tumour size, cm — mean ± SD	2.7 ± 1.9 (n=18)	1.4 ± 0.6 (n=3)
Treatment		
Systemic chemotherapy	16 (76.2%)	3 (100.0%)
Radiotherapy	7 (33.3%)	2 (66.7%)
Mastectomy	2 (9.5%)	0 (0.0%)
Outcomes		
Progression / relapse	8 (38.1%)	3 (100.0%)
Death (any cause)	11 (52.4%)	2 (66.7%)
Disease-related death	2 (9.5%)	1 (33.3%)
Alive without evidence of disease	5 (23.8%)	0 (0.0%)
Alive with disease	5 (23.8%)	1 (33.3%)
Median observation, months (range)	99.1 (7.9–217.6)	66 (50.3–85.4)
Median time to progression, months	53.1	33.8

Primary breast involvement was defined according to Wiseman–Liao criteria. Given the small number of secondary cases, no statistical comparison was performed and the differences shown are descriptive only.

Confidence intervals are wide throughout, reflecting the number of events, and these estimates should be interpreted as descriptive of this cohort rather than as generalizable benchmarks.

## Discussion

This study presents a retrospectively characterized single-institution European cohort of HBM, among the larger series reported to date, comprising 24 patients accrued over 15.5 years across the full histological spectrum. We note that larger series exist, including the 58-patient cohort of Guilbert et al. and the multicenter IELSG series. The main findings are: (i) DLBCL is the predominant subtype, consistent with existing literature; (ii) all diagnoses were achieved by image-guided core needle biopsy, supporting the diagnostic sufficiency of this approach; (iii) no positive ER or PR result was identified among cases with available pathology documentation; this finding may support hematological lineage within an appropriate marker panel but is not independently discriminatory; (iv) mastectomy was avoidable in 91.7% of patients; and (v) overall and disease-specific survival diverge markedly, indicating that most deaths in this cohort were not attributable to the hematologic disease.

The incidence and subtype distribution in our cohort closely mirror those of the largest contemporary reference series. Guilbert et al. reported DLBCL as the most prevalent subtype (50%) in a series of 58 HBM patients from a major US academic center, followed by marginal zone lymphoma and follicular lymphoma (6). Our series shows a similar hierarchy, with a relatively higher proportion of MALT lymphoma (20.8%), which may reflect referral patterns at our center. The absence of myeloma in our cohort, despite its known capacity for soft-tissue and extramedullary infiltration in advanced disease, likely reflects the predominantly early-stage and localized disease pattern of our series. Neither T-cell lymphomas nor plasma cell neoplasms were represented in our series. Their absence probably reflects both the referral pattern of our center and the limited size of the cohort, and it means that our observations cannot be extended to those entities.

A central diagnostic challenge in HBM is its morphological overlap with primary breast carcinoma, particularly invasive lobular carcinoma, which shares the single-file infiltrative pattern seen in small B-cell lymphomas and leukemic infiltrates (4, 5). In our series, no positive ER or PR result was identified among the cases with available pathology documentation, although a reliable testing denominator could not be retrospectively reconstructed. This finding is consistent with hematologic lineage, but we would not present it as a discriminator: it does not distinguish HBM from triple-negative or metaplastic breast carcinoma, and hormone receptor expression has itself been reported in lymphoma (4). Its value lies in supporting a lineage assignment that rests primarily on the B-cell marker panel.

This finding reinforces the importance of incorporating a lymphoma marker panel (CD20, CD3, CD10, BCL2, BCL6) in the immunohistochemical workup of any atypical breast biopsy where the morphology does not unequivocally indicate epithelial origin. Regarding surgical management, both mastectomies performed in our cohort were patient-driven decisions made after full disclosure of the hematological diagnosis—an important nuance that must be clearly communicated during multidisciplinary team discussions to avoid misclassifying these cases as evidence of surgical need.

The mode of detection in our series was heterogeneous. Eleven cases (45.8%) were detected by mammography, 8 (33.3%) presented as a palpable breast mass, and 5 (20.8%) were identified incidentally or during follow-up imaging (**Table 1** and **Figure 1B**). Systematic imaging descriptors were not recorded uniformly across the accrual period and could not be reliably analyzed. Therefore, our data do not support the attribution of a specific mammographic, ultrasonographic or magnetic resonance imaging pattern to HBM. Only 2 patients (8.3%) had documented B symptoms, indicating that most patients in this cohort presented without systemic manifestations. These findings emphasize the importance of histopathological assessment in the diagnostic pathway.

From a therapeutic standpoint, HBM should be managed according to its specific hematological subtype, with breast surgery reserved for exceptional circumstances rather than considered a standard therapeutic approach (7, 9). Treatment in our cohort was heterogeneous and reflected the histological diagnosis, disease extent, patient characteristics and changes in clinical practice across the study period. Systemic treatment was administered to 19 patients (79.2%), while 9 patients (37.5%) received breast-directed radiotherapy, including 5 patients with DLBCL (**Table 2**). Given the small sample size, retrospective design and absence of detailed treatment and response information, our data cannot establish the comparative efficacy of particular systemic regimens or radiotherapy strategies. No patient was documented as having received prophylactic CNS-directed treatment. However, CNS-IPI components and laterality were not consistently available, preventing any assessment of the appropriateness or potential benefit of CNS prophylaxis.

The most informative outcome finding in this cohort is the divergence between overall and disease-specific survival. Median overall survival was 115.7 months, whereas disease-specific survival was not reached and was estimated at 86.8% at 15 years. Only 3 of 13 deaths were attributable to disease progression. In a cohort with a median age of 60 years, a range extending to 93, and follow-up of up to 18 years, competing mortality dominates the overall survival curve. We therefore do not compare our overall survival figures with published series for primary breast DLBCL (8, 13), since those comparisons would conflate disease-driven and competing mortality. In the CLL/SLL subgroup, both deaths were from unrelated causes and no progression event was documented; we no longer interpret these deaths as evidence of an inexorably progressive natural history, as the original version of this manuscript did. The clinical implication of this pattern is that treatment intensity in HBM should be calibrated to competing risks as much as to the hematologic diagnosis itself.

Our study carries several inherent limitations. The retrospective single-center design limits generalizability and introduces potential selection bias. The total sample of 24 patients, while among the largest for this rare entity in a European single-institution report, does not permit multivariable analysis or reliable statistical subgroup comparison. Molecular and genomic data—including MYC/BCL2/BCL6 rearrangement status, cell-of-origin classification for DLBCL, and next-generation sequencing profiling—were not systematically available, precluding correlation of biological features with outcomes. Treatment heterogeneity across the 15-year study period, spanning substantial evolution in both diagnostic pathology and systemic therapy, may introduce temporal confounding that cannot be formally corrected for in this analysis. Notwithstanding these limitations, the breadth of subtype representation, the completeness of the clinical dataset, and the length of follow-up provide a meaningful contribution to the sparse evidence base for this disease.

Several further limitations warrant emphasis. Our original working database proved to have incomplete mortality ascertainment, which was corrected by a systematic review of clinical records for the present revision; this experience argues for caution in interpreting retrospective single-center outcome data generally. Cause of death was assigned from the clinical record and was not adjudicated independently, and it was not documented in one patient. Prognostic indices, staging modality and molecular data were not retrievable for the full cohort. Treatment detail, including radiotherapy dose and technique and formal response assessment, was not uniformly recorded across the accrual period.

## Conclusions

This study describes the clinicopathological characteristics, management and long-term outcomes of a rare and histologically heterogeneous group of hematological malignancies involving the breast. Image-guided core needle biopsy enabled diagnosis without breast resection in 22 of 24 patients, underscoring the importance of appropriate histopathological and hematolymphoid lineage assessment when evaluating atypical breast lesions.

After a median observed duration of 86.9 months, 13 patients had died, but only 3 deaths were attributed to progression of the hematological malignancy. The resulting divergence between overall and disease-specific survival highlights the importance of competing causes of mortality when interpreting outcomes in this cohort. Treatment should be directed according to histological subtype and individual clinical context, while breast surgery should be reserved for exceptional circumstances rather than considered a standard therapeutic approach.

No positive ER or PR result was identified among cases with available pathology documentation. This finding may support hematological lineage within an appropriate immunohistochemical panel but cannot independently distinguish these malignancies from primary breast carcinoma. Given the retrospective, single-center design, small sample size and heterogeneous histological composition, the findings should be interpreted descriptively. Larger multicenter studies with systematic staging, molecular characterization and standardized outcome assessment are required.

## Data Availability

The raw data supporting the conclusions of this article will be made available by the authors, without undue reservation.
