# Supplementary material for: Hematologic breast malignancies: a single-institution cohort study of 24 patients diagnosed between January 2008 and June 2023, with clinicopathological and outcome analysis
Source: Front Oncol. 2026 Oct 21;16:1925669. doi: 10.3389/fonc.2026.1925669 (PMC13730824; doi:10.3389/fonc.2026.1925669)
Supplement: Supplementary file 1 [file DataSheet1.docx]

**Supplementary Figure 1**

Representative histopathological images of a core needle biopsy of an axillary lymph node showing infiltration by diffuse large B-cell lymphoma. Immunostaining at ×20 magnification demonstrates: (A) strong and uniform CD20 positivity confirming mature B-cell phenotype; (B) BCL6 expression; (C) MUM1 positivity; and (D) elevated Ki-67 index indicating a high proliferative rate.
